# Relationships between the Circadian System and Alzheimer's Disease-Like Symptoms in Drosophila

**DOI:** 10.1371/journal.pone.0106068

**Published:** 2014-08-29

**Authors:** Dani M. Long, Matthew R. Blake, Sudeshna Dutta, Scott D. Holbrook, Joanna Kotwica-Rolinska, Doris Kretzschmar, Jadwiga M. Giebultowicz

**Affiliations:** 1 Department of Integrative Biology, Oregon State University, Corvallis, Oregon, United States of America; 2 Oregon Institute of Occupational Health Sciences, Oregon Health and Science University, Portland, Oregon, United States of America; McGill University, Canada

## Abstract

Circadian clocks coordinate physiological, neurological, and behavioral functions into circa 24 hour rhythms, and the molecular mechanisms underlying circadian clock oscillations are conserved from *Drosophila* to humans. Clock oscillations and clock-controlled rhythms are known to dampen during aging; additionally, genetic or environmental clock disruption leads to accelerated aging and increased susceptibility to age-related pathologies. Neurodegenerative diseases, such as Alzheimer's disease (AD), are associated with a decay of circadian rhythms, but it is not clear whether circadian disruption accelerates neuronal and motor decline associated with these diseases. To address this question, we utilized transgenic *Drosophila* expressing various Amyloid-β (Aβ) peptides, which are prone to form aggregates characteristic of AD pathology in humans. We compared development of AD-like symptoms in adult flies expressing Aβ peptides in the wild type background and in flies with clocks disrupted via a null mutation in the clock gene *period* (*per^01^*). No significant differences were observed in longevity, climbing ability and brain neurodegeneration levels between control and clock-deficient flies, suggesting that loss of clock function does not exacerbate pathogenicity caused by human-derived Aβ peptides in flies. However, AD-like pathologies affected the circadian system in aging flies. We report that rest/activity rhythms were impaired in an age-dependent manner. Flies expressing the highly pathogenic arctic Aβ peptide showed a dramatic degradation of these rhythms in tune with their reduced longevity and impaired climbing ability. At the same time, the central pacemaker remained intact in these flies providing evidence that expression of Aβ peptides causes rhythm degradation downstream from the central clock mechanism.

## Introduction

The circadian clock coordinates daily physiological, neurological, and behavioral rhythms which maintain temporal homeostasis. The molecular clock mechanism is highly conserved from *Drosophila* to mammals and consists of a two-arm negative feedback loop. The positive arm proteins in *Drosophila* are CLOCK (CLK) and CYCLE (CYC) which form a dimer driving the transcription of the *period (per)* and *timeless (tim)* genes, which encode the negative arm clock proteins. PER and TIM proteins dimerize in the cytoplasm and translocate into the nucleus where PER suppresses transcriptional activity of CLK and CYC proteins. Flies with null mutations in any one of these core clock genes have abolished clock function and are behaviorally arrhythmic [1].

The significance of circadian regulation for organismal health is emerging from studies showing that genetic or environmental disruption of the circadian system leads to premature aging and age-related pathologies. For example, mice lacking the clock protein BMAL1 (homolog of fly CYC protein) show several symptoms of aging [2], [3], and loss of BMAL1 in the brain may lead to neurodegeneration [4]. In flies, a null mutation in the clock gene *per* leads to higher accumulation of ROS, protein carbonyls, and peroxidated lipids during aging [5], [6], suggesting that antioxidant defenses are compromised by the loss of clock function. We also reported recently that disruptions in clock function in flies accelerated aging in neurodegeneration-prone *sniffer* and *swiss cheese* mutants [6].

Observations that mutations in clock genes may accelerate neurodegeneration opens the question whether clock genes play any roles in the most prevalent of all neurodegenerative diseases, namely Alzheimer disease (AD). Links between AD and the circadian system are suggested by common observations that an early symptom of AD in humans is fragmented sleep/wake patterns with increasing nighttime activity and daytime naps [7], [8], and that preclinical changes in rest-activity parameters serve as significant predictors of subsequent dementia [9]. A study on postmortem brains of AD patients revealed altered timing of circadian gene expression in various brain regions suggesting circadian desynchrony [10]. Additionally, impaired rest/activity rhythms have been observed in experimental AD model mice [11], [12]. While these data support weakening of circadian rhythms in AD, it is not clear whether AD-related neurodegeneration disrupts the circadian clock or conversely, disruption of the clock contributes to AD progression, or both.

One of the major factors implicated in AD pathogenesis is the aggregation of amyloid β (Aβ) protein fragments in the brain, resulting in neuronal cell death [13]. Amyloid β is a peptide produced by cleavage of the Amyloid Precursor Protein (APP) by β- and γ-secretases. Depending on the cleavage site, fragments of 40 (Aβ_40_) or 42 (Aβ_42_) amino acids are produced; the longer Aβ_42_ is more prone to form amyloid plaques in aging individuals. The arctic form of Aβ_42_ (Aβ_42_arc) causes fragments that are even more pathogenic due to a mutation (E22G) that causes the fragments to aggregate more readily [14].

*Drosophila* models of AD have been developed in which human Aβ_40_, Aβ_42_, or Aβ_42_arc can be directly expressed in the nervous system [15]. These flies show progressive locomotor deficits, vacuolization in the brain and premature death with the severity of the symptoms being proportional to the pathogenicity of human Aβ fragments [15]–[17]. In particular, expression of the arctic mutation induces increased amyloid aggregation and accelerated rates of neurodegeneration [17].

In this work, we investigated AD model flies with a normal or disrupted circadian system to address three main questions: 1) Does arrhythmicity caused by the loss of the core clock gene *period* accelerate AD-like phenotypes in the fly model? 2) Does AD progression alter circadian locomotor activity rhythms in flies? 3) Is the central pacemaker functional in aging AD-model flies? In order to address these questions, we used transgenic *Drosophila* expressing human Aβ_40_, Aβ_42_, or Aβ_42_arc fragments [15] under the control of the pan-neuronal driver *elav*-GAL4 in clock-competent or clock-disrupted *per^01^* backgrounds. These model flies enable us to tease apart putative interactions between the circadian clock and AD symptom progression.

## Methods and Materials

### Fly rearing and crosses

*Drosophila melanogaster* were reared on diet containing 1% agar, 6.25% cornmeal, 6.25% molasses, and 3.5% Red Star yeast at 25°C. Flies were entrained to 12-hour light:dark (LD, 12∶12) cycles (with an average light intensity of ∼1500 lx). All experiments were performed on mated male flies of different ages, as specified in the results. To obtain AD model flies expressing human Aβ peptides, transgenic males carrying different UAS-Aβ constructs [15] were crossed to females carrying the pan-neuronal driver *elav*-GAL4 in the *per^+^* or *per^01^* (null-mutant) background. For controls, *elav* or *elav-per^01^* females were crossed to UAS-GFP males.

### Longevity assay

Lifespan was measured using at least two bioreplicates of 4 cohorts of 50 mated males. Males of a given genotype were housed in 8 oz. round bottom polypropylene bottles (Genesee Scientific, San Diego, CA) inverted on 60 mm Falcon Primaria Tissue culture dishes (Becton, Dickinson and Company) containing 15 µL of diet. Flies were tapped to the bottom of the bottles without anesthesia for diet exchange and mortality recording every 2–3 days.

### Rapid iterative negative geotaxis (RING)

Vertical climbing abilities of male flies were measured using the RING assay following an established protocol [18]. For each genotype tested, 3 groups of 25 flies were transferred without anesthesia into empty vials and placed in the RING apparatus. The apparatus was tapped 3 times in rapid succession to initiate a negative geotaxis response. Movements of the flies in the tubes were videotaped, and digital images were captured at the 4 second mark after the tapping was completed. Using ImageJ software (NIH), the height climbed by each fly was calculated in centimeters for the snapshot at the 4 sec interval. The average of 5 successive trials, separated by 30 second rest periods, was used to calculate the performance of the flies in a single vial.

### Neurodegeneration

Flies of each genotype were aged to 20 days and heads were processed as previously described [19], [20]. Briefly, heads were cut in 7 µm serial sections, the paraffin was removed in SafeClear (Fisher Scientific), sections were embedded in Permount, and analyzed with a Zeiss Axioscope 2 microscope using the auto-fluorescence caused by the eye pigment (no staining was used). Experimental and control flies were put next to each other in the same paraffin block, cut, and processed together. Microscopic pictures were taken at the same level of the brain, the vacuoles (identified by being unstained and exceeding 50 pixels in size) were counted and the vacuolized area was calculated using our established methods [19], [20].

### Activity rhythms

Locomotor activity patterns were monitored as described [21] using the Trikinetics Drosophila Activity Monitoring System (DAMS; Waltham, MA). Individual flies were recorded for 3 days in LD conditions and then for 7 days in constant darkness (DD). Average activity graphs were generated using GraphPad based on activity data from three consecutive 24-h periods of 12∶12 LD at 25°C. ClockLab software (Actimetrics, Coulbourn Instruments) was used to generate actograms and periodograms for the analysis of free running rhythms including period length in DD. Overall rhythm strength of individual flies was determined using a Fast Fourier Transformation (FFT) and averaged for each genotype and age. Flies with a FFT value greater than 0.04 and/or a periodogram with a peak that breaks the significance line were considered rhythmic.

### Immunocytochemistry

To determine the functional integrity of the central clock, we measured levels of the clock protein PER in brain whole-mounts. Co-staining with PDF was used to identify specific central clock cells. Flies over-expressing Aβ_42_arc via the pan-neuronal driver *elav*-GAL4 were tested along with driver (*elav*-GAL4/+) or responder (UAS-Aβ_42_arc/+) controls at days 5 and 15. Samples were collected at Zeitgeber time (ZT) 22 and ZT10, which correspond, to high and low levels of PER protein in wild type flies, respectively. Whole flies were fixed in freshly prepared 4% paraformaldehyde in Phosphate Buffer Saline (PBS) with 0.1% Triton-X100 (PBS-T 0.1%) for 30 min. Fly brains were then dissected in PBS-T 0.1% and placed in wells containing PBS-T 0.1%. After dissection, the brains were fixed in 4% paraformaldehyde for 10 min, rinsed with PBS with 0.5% Triton X100 (PBS-T 0.5%), and blocked overnight in 5% Normal Goat Serum (NGS) in PBS-T 0.5%. Brains were then incubated for 48 hours in primary 1∶500 mouse nb33 monoclonal anti-PDF (Developmental Studies Hybridoma Bank) and 1∶10,000 pre-absorbed rabbit anti-PER (gift from Dr. R. Stanewsky), rinsed 6 times in PBS-T 0.5% and incubated overnight in secondary Alexa Fluor 555 anti-mouse (1∶500) and Alexa Fluor 488 anti-rabbit (1∶1000) (Life Technologies). Samples were rinsed 6 times with PBS-T 0.5% and mounted on microscope slides in Vectashield mounting media with DAPI (Vector Laboratories, Burlingame, California). Images were taken with an Olympus FV300 confocal microscope with all laser parameters held constant throughout. PER levels were evaluated by measuring the fluorescence intensity in pacemaker cell nuclei after converting the mean level of fluorescence to the Mean Gray Value that was quantified using Fiji software [22].

### Statistical analyses

Lifespan graphs were plotted using survival curves and statistical significance between the curves determined using the Log-Rank (Mantel-Cox) test (GraphPad Prism v5.0;GraphPad Software Inc. San Diego, CA). Statistical significance in climbing ability across different ages and genotypes was determined using two-way ANOVA with Bonferroni's post-test using GraphPad Prism5 software. For neurodegeneration, average vacuole size and count were completed using Photoshop software with statistical significance determined by one-way ANOVA and Dunett's post-test using Graphpad Prism5 software. Statistical significance for average FFT and intensity of PER staining was calculated by unpaired t-test with Welch's correction using GraphPad Prism5.

## Results

### Lifespan reduction caused by Aβ peptides is not exacerbated by the by loss of the clock gene *period*

**Figure 1:**
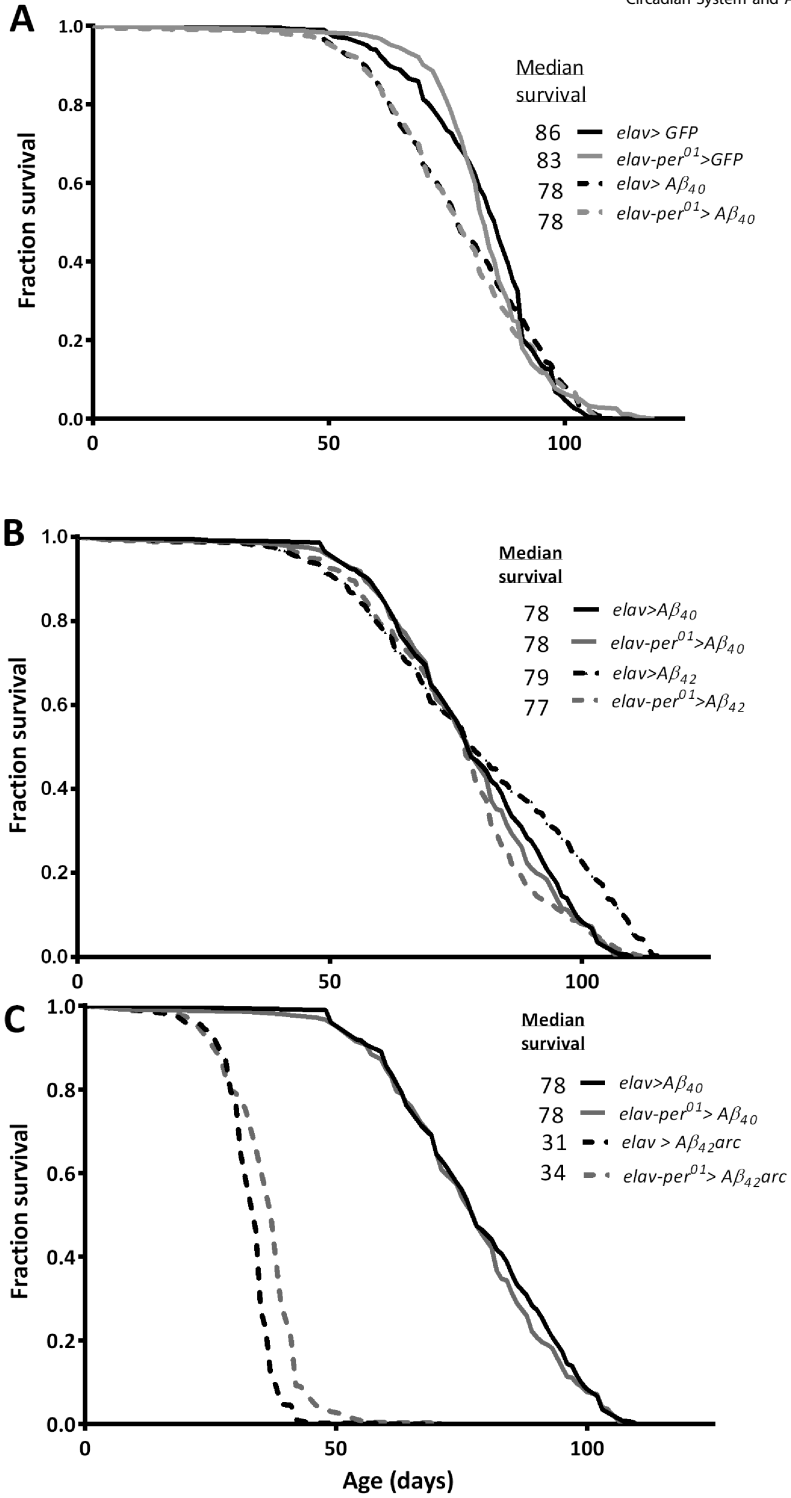
Longevity of flies expressing Aβ peptides. Survival curves and median lifespans of flies expressing GFP or Aβ_40_ (A), Aβ_40_ or Aβ_42_ (B), and Aβ_40_ or Aβ_42_arc (C). At least 400 individuals were used for each genotype tested. According to Mantel-Cox log rank test flies expressing Aβ_42_arc had significantly shortened lifespan (p<0.0001) relative to Aβ_40_ controls irrespective of whether their clock was disrupted by *per*-null mutation or not.

To determine whether disruption of the clock affects longevity in AD model flies, we compared the lifespan of flies expressing various Aβ peptides in wild type (*per^+^*) and clock-disrupted (*per^01^*) mutant backgrounds. Pan-neuronal expression of Aβ_40_ peptides did not significantly shorten lifespan compared to control *elav>gfp* flies expressing GFP, therefore, Aβ_40_ was considered as another control. Expression of Aβ_42_ in flies with normal or disrupted clocks had no effect on lifespan (Fig 1A–B). Dramatic lifespan shortening (p<0.0001) was observed in flies expressing the strongly pathogenic Aβ_42_arc but the loss of *per* function did not worsen this phenotype (Fig 1C).

### Flies expressing Aβ_42_arc show similar motor decline and neurodegeneration in clock-positive and clock-disrupted backgrounds

**Figure 2:**
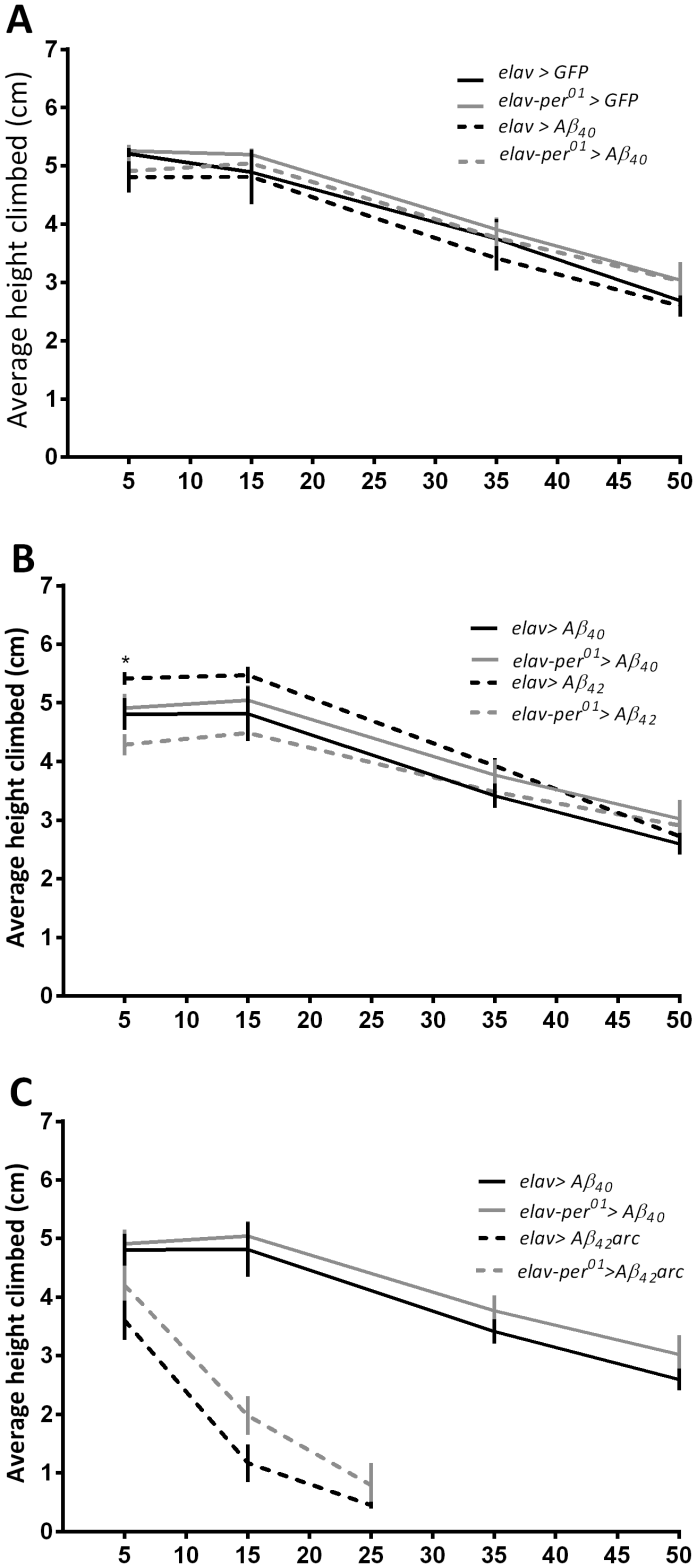
Motor decline is proportional to pathogenicity of Aβ peptides with no consistent effects due to clock disruption. Average vertical height climbed was measured in flies expressing GFP or Aβ_40_ (A) and Aβ_40_ or Aβ_42_ (B) on days 5, 15, 35, and 50. C) Aβ_40_ compared to Aβ_42_arc was tested only on days 5, 15, and 25 due to increased mortality. Statistical significance calculated using 2-way ANOVA is shown in Table S1.

To investigate effects of *per^01^* on motor impairments associated with Aβ peptides, we monitored age-specific climbing performance using RING assays. There was no significant difference in the average distance climbed between *elav>Aβ_40_* or *elav-per^01^>Aβ_40_* flies, and their respective controls expressing GFP (Fig. 2A). Interestingly, the climbing ability was significantly impaired (p<0.05) at day 5 in flies expressing Aβ_42_ in the *per^01^* background compared to those with normal clock. However, this difference was not observed in older flies, rather, all genotypes showed similar climbing performance on days 15, 35, and 50 (Fig 2B). Young 5-days old flies expressing the most pathogenic Aβ_42_arc peptide showed a modest reduction in vertical climbing ability relative to *elav>Aβ40* controls on day 5 (Fig 2C). Climbing ability rapidly declined in 15-days old *elav>Aβ_42_arc* and *elav-per^01^>Aβ_42_arc* flies compared to their respective controls (p<0.0001) and was essentially lost in a few flies that survived for 25-days (Fig 2C).

**Figure 3:**
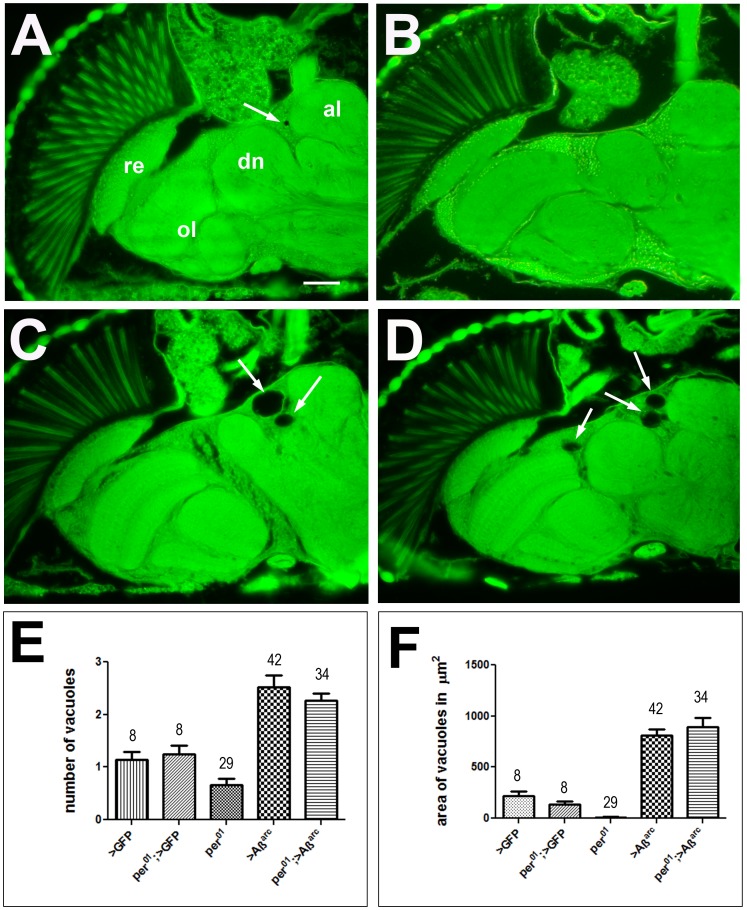
Flies expressing Aβ_42_arc show increased brain vacuolization compared to age-matched controls regardless of *per* status. Brain slices of *elav >gfp* (A), *per^01^* (B), *elav>Aβ_42_arc* (C), and *elav-per^01^>Aβ_42_arc* (D) at age 20 days. Arrows point to vacuoles. E) Mean number of vacuoles in each genotype. F) Mean vacuole area in µm^2^ in each genotype. Numbers above bars indicate number of brain hemispheres examined and the SEM is indicated. re = retina, ol = optic lobes, dn =  deutocerebral neuropil, al = antennal lobe. Bar = 25 µm.

We reported recently that the loss of *per* function accelerated brain vacuolization in neurodegeneration prone fly mutants [6]. Therefore, we examined brain health in 20-days old *elav>Aβ_42_arc* and *elav-per^01^>Aβ_42_arc* flies and their respective controls. Control *elav>gfp* flies often showed a single vacuole which was however quite small (arrow, Fig. 3A). Similarly, *elav-per^01^>gfp* (not shown) and *per^01^* (Fig. 3B) control flies showed some small vacuoles. Age-matched *elav>Aβ_42_arc* flies showed a significant increase in vacuole number as well as size (p<0.01 to controls for both). However, the average number and size of vacuoles was not further increased when *elav>Aβ42arc* was combined with *per^01^* (Fig 3D). A quantification of this phenotype is shown in figure 3E and F.

### Daily locomotor activity rhythms are impaired in aging flies expressing different Aβ peptides

**Figure 4:**
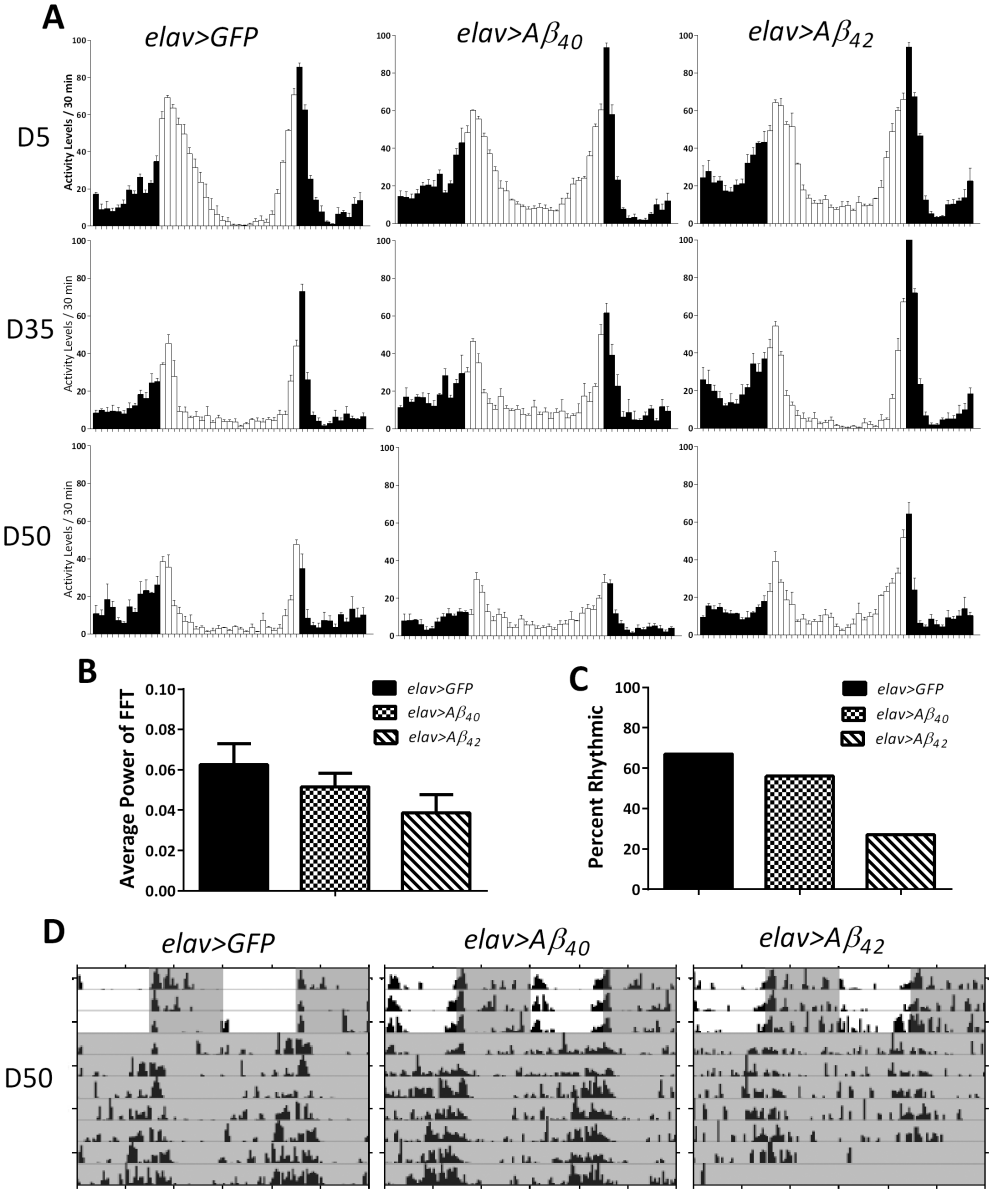
Effects of Aβ expression on age-dependent locomotor activity patterns. A) Panels depict the average daily locomotor activity for Aβ_40_, Aβ_42_, and GFP controls at ages 5, 35, and 50 days based on three consecutive 24-h periods in 12∶12 LD. Vertical bars represent activity recorded in 30- min bins during times of lights on (white bars) or off (black bars). B) Free-running rhythm strength based on average FFT at age 50 days in flies expressing GFP, Aβ_40_ or Aβ_42_. FFT determined during 6 days of DD C) Percent of rhythmic flies was reduced in Aβ_42_ expressing flies compared to GFP and Aβ_40_ expressing ones at age 50 days. D) Representative actograms of individual flies of Aβ_40_, Aβ_42_, and GFP expressing flies at age 50 days. Gray shading indicates lights off.

To test whether circadian rhythms are affected by amyloidogenic peptides, daily locomotor activity patterns were compared between *elav>gfp*, *elav>Aβ_40_*, and *elav>Aβ_42_* flies that were 5, 35, and 50 days old. Average daily activity patterns in LD were similar in all genotypes, showing morning and evening activity peaks that were attenuated with age (Fig 4A). Analysis of locomotor activity in constant darkness (DD) revealed that the endogenous activity rhythms were not significantly impaired in 5- or 35-days old *elav> Aβ_42_* or *elav>Aβ_40_* flies compared to age-matched *elav>gfp* controls (Table 1). However, 50-days old flies expressing Aβ_42_ exhibited a substantial decrease in rhythm strength (Fig 4B) and the fraction of flies that remained rhythmic was only 27% compared to 67% in controls (Fig 4 C and Table 1). Representative examples of activity rhythms in 50-day old individuals are shown in Fig 4D.

**Table 1:** Effects of human Aβ peptides on circadian locomotor activity.

Age	Genotype	n	% Rhythmicity (Strong + Weak)	Rhythm Strength (Average FFT ± SEM)	Period (DD)
**Day 5**	elav-gal4 > UAS-GFP	37	78% (54%+24%)	0.107±0.012	23.43
	elav-gal4 > UAS-Aβ_40_	25	60% (48%+12%)	0.069±0.009	23.59
	elav-gal4 > UAS-Aβ_42_	24	75% (54%+21%)	0.077±0.010	23.45
**Day 35**	elav-gal4 > UAS-GFP	30	50% (27%+23%)	0.049±0.006	23.64
	elav-gal4 > UAS-Aβ_40_	12	83% (58%+25%)	0.070±0.009	23.73
	elav-gal4 > UAS-Aβ_42_	10	60% (20%+40%)	0.047±0.008	23.73
**Day 50**	elav-gal4 > UAS-GFP	15	67% (47%+20%)	0.063±0.011	24.1
	elav-gal4 > UAS-Aβ_40_	25	56% (36%+20%)	0.052±0.007	24.02
	elav-gal4 > UAS-Aβ_42_	14	27% (20%+7%)	0.038±0.009	24.31
**Day 5**	elav-gal4 > UAS-GFP	37	78% (54%+24%)	0.107±0.012	23.43
	elav-gal4 > UAS-Aβ_42_arc	46	20% (11%+9%)	0.028±0.005****	23.62
**Day 15**	elav-gal4 > UAS-GFP	23	70% (18%+52%)	0.049±0.005	23.35
	elav-gal4 > UAS-Aβ_42_arc	26	19% (4%+15%)	0.021±0.004***	23.94

Flies with a FFT value less than 0.04 were considered arrhythmic, 0.04–0.08 were considered weakly rhythmic, and greater than 0.08 were considered strongly rhythmic. Average FFT values for different genotypes at the same age were compared by unpaired t-test using Welch's correction. (***p = 0.0001 ****p>0.0001).

**Figure 5:**
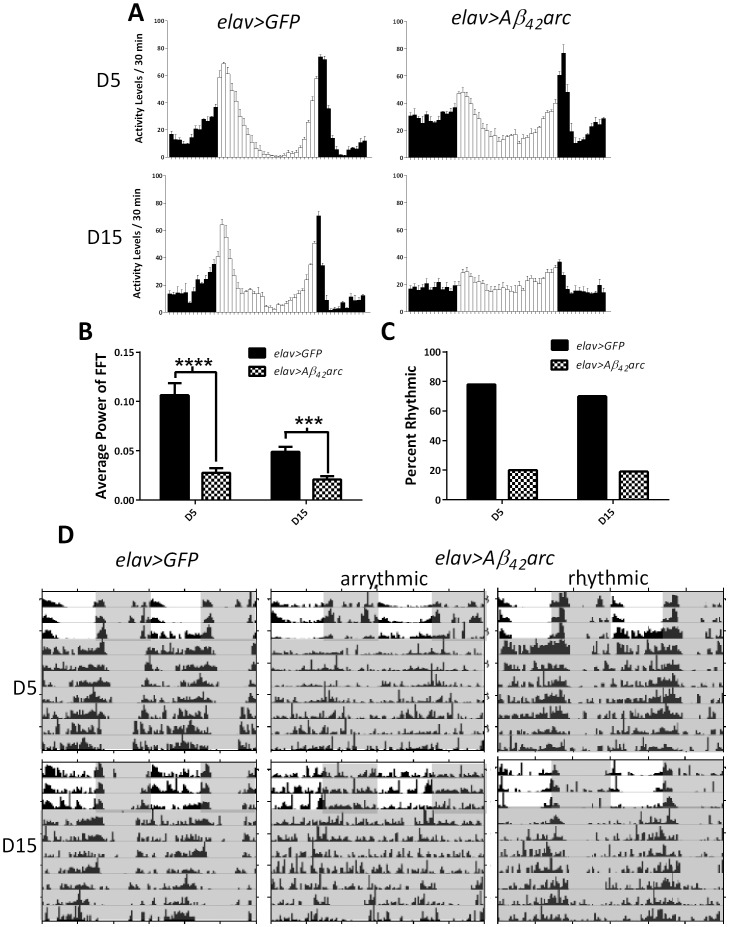
Locomotor activity becomes non-rhythmic in flies expressing Aβ_42_arc. A) Panels depict the average daily locomotor activity in 5- and 15-day old flies. Graphs are generated based on activity data from three consecutive 24-h periods of 12∶12 LD. Vertical bars represent activity recorded in 30- min bins during times of lights on (white bars) or off (black bars) of Aβ_42_arc expressing flies and controls. B) Average rhythm strength based on FFT determined during 6 days in DD in 5- and 15-days old flies with Aβ_42_arc expression and control flies. Average FFT is significantly lower in experimental flies at day 5 and 15 (p<0.0001 and p = 0.0001, respectively). C) Percent of rhythmic flies is substantially lower when Aβ_42_arc is induced than in controls at age 5 and 15 days. Individuals with FFT scores over .04 and/or a period that breaks the significance line were considered rhythmic. D) Representative actograms of individual flies of genotypes *elav>Aβ_42_arc* (both rhythmic and arrhythmic) and *elav>gfp* controls at ages 5 and 15 days. Gray shading indicates lights off.

We also measured locomotor activity of flies expressing Aβ_42_arc but due to their decreased survival (Fig 1), we tested them at ages 5 and 15 days only. While, control *elav>gfp* flies showed strong bimodal activity rhythms in LD with morning and evening peaks preceded by anticipatory mobility increase, *elav>Aβ_42_arc* flies showed an impaired activity rhythm with a reduced morning peak already on day 5 (Fig. 5A). Loss of rhythmicity was even more severe in 15-days old *elav>Aβ_42_arc* flies such that the activity was distributed evenly around the clock without distinct morning and evening peaks or nighttime rest (Fig. 5A). Analysis of activity in DD revealed that the average rhythm strength was significantly decreased at both day 5 and 15 (Fig. 5B) and the percentage of rhythmic individuals was markedly reduced in Aβ_42_arc expressing flies (Fig. 5 C; Table 1). Most of these flies were active around the clock (Fig. 5D, middle panels) and the remaining flies showed weaker rhythms (Fig. 5D, right panels) compared to well-defined rhythms recorded in age-matched controls (Fig. 5D, left panels).

### Rhythms in PER cycling continue in lateral neurons of elav>Aβ_42_arc flies

**Figure 6:**
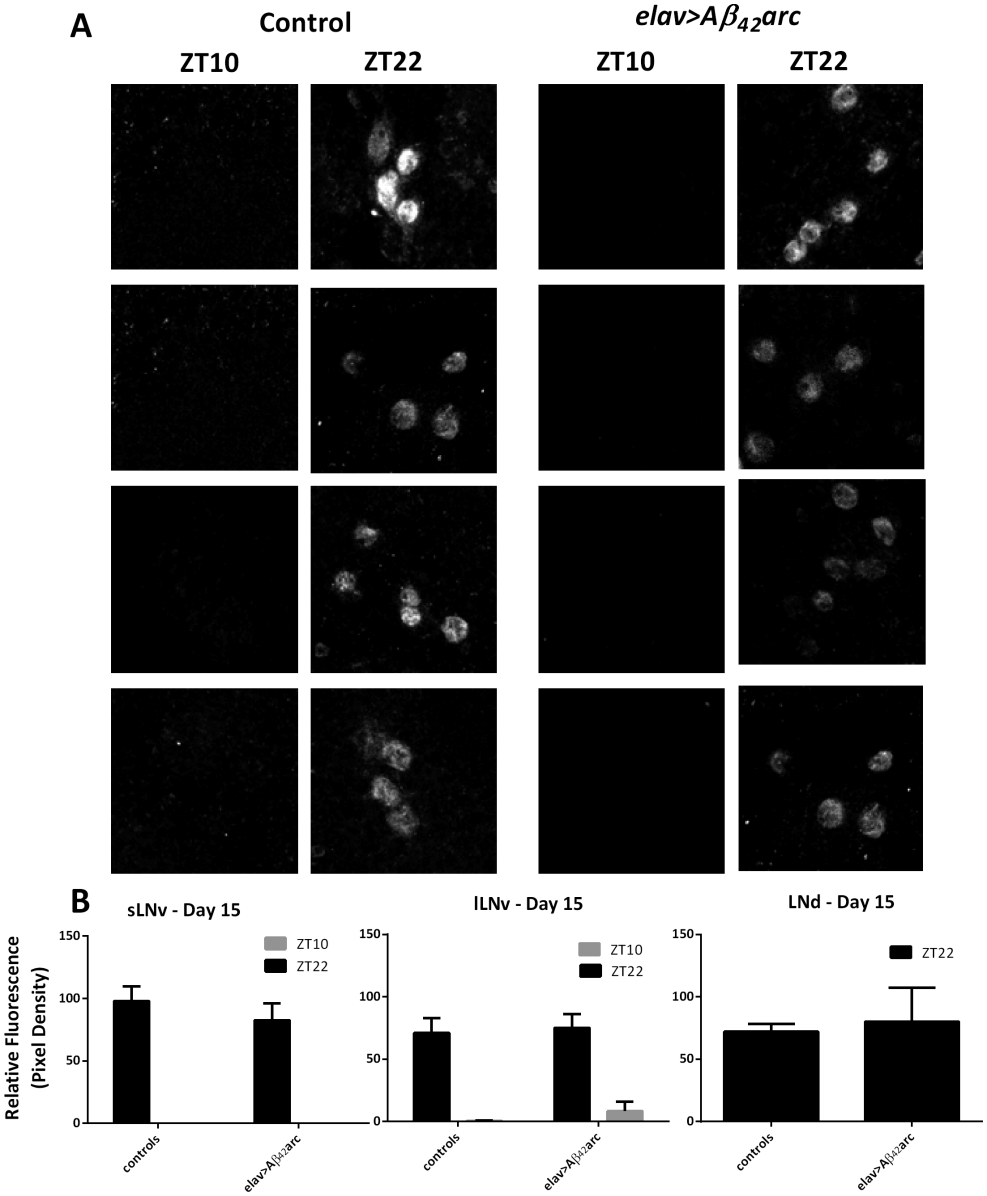
Immunocytochemistry shows that PER oscillations are normal in Aβ_42_arc expressing flies in 12∶12LD. A) Images of s-LN_v_ and l-LN_v_ in elav>Aβ_42_arc and controls at age 5 and 15 days at ZT10 and ZT22. Brains were stained for PDF (not shown) to identify clock neurons at ZT10 and ZT22. Pictures show levels of nuclear PER in these neurons. B) Graphical representation of relative fluorescence based on pixel density in specified neuron groups at ZT10 and ZT22. LN_d_ could not be identified at ZT10, therefore PER signal is shown only at ZT22. To increase sample size, two controls *elav-*GAL4/+ and UAS-*Aβ_42_arc*/+ were combined in statistical calculations.

The loss of behavioral rhythms in *elav>Aβ_42_arc* flies could be caused by disruptions in the central clock mechanism or in the output pathways. The central clock is comprised of several groups of neurons that contribute to the control of behavioral rhythms [23]. These include two groups of neurons expressing the pigment dispersing factor (PDF); the small lateral neurons (s-LN_v_) that are critical for free-running activity rhythms and the large lateral neurons (l-LN_v_) important in overall arousal, as well as the PDF-negative dorsal lateral (LN_d_) neurons and the 5^th^ s-LN_v_ neuron. To investigate whether the loss of behavioral rhythms in flies expressing Aβ_42_arc was caused by defects in the central clock mechanism, we measured PER expression in these neurons using immunocytochemistry. PDF-expressing s-LN_v_ and l-LN_v_ were identified using an anti-PDF antibody. We observed strong PDF signals in both control and *elav>Aβ_42_arc* flies, furthermore, the morphology and number of PDF-positive neurons (4+4) were not altered in *elav>Aβ_42_arc* flies (not shown). We determined that the PER protein shows oscillations in these pacemaker neurons of *elav>Aβ_42_arc* flies similar to controls with high levels of nuclear PER at ZT22 and absence of the signal at ZT10 (Fig 6A). This suggests that the pattern normally found in functional clock cells is not disturbed in *elav>Aβ_42_arc* flies, although they were behaviorally arrhythmic. Quantification of the signals confirmed a lack of significant differences in relative PER fluorescence between control and experimental flies (Fig. 6B).

## Discussion

Associations between AD and impaired daily rhythms are well documented in humans, yet the causes and consequences of AD-related loss of circadian sleep/activity rhythms have not been teased apart. One of the unanswered questions is whether age-related decline of the circadian system contributes to AD progression. This study tested directly whether total arrhythmia caused by mutation in the core clock gene *per* would exacerbate AD-like phenotypes observed in an AD fly model. We determined that premature death, progressive locomotor deficits, and vacuolization in the brain occurred with similar timing and intensity in flies with genetically disrupted clock mechanism as in control flies. Consistent with previous reports [15], [17], the severity of symptoms was proportional to the pathogenicity of the expressed human Aβ fragments. However, within each genotype, symptoms in clock-deficient flies were similar to those in clock-competent flies. While our data show that disruption of the clock via removal of the core clock repressor PER does not exacerbate AD symptoms, we cannot exclude that disabling the positive clock arm could be more detrimental. A recent report showed that loss of the positive element BMAL1 causes brain neurodegeneration in mice [4]. We previously demonstrated that the loss of *per* accelerates death, locomotor impairments, and brain vacuolization in neurodegeneration-prone *sniffer* and *swiss cheese* fly mutants [6]. However, we do not know the underlying molecular mechanism that mediates this effect. The AD model used here is based on the expression of human Aβ peptides, which have been reported to accumulate into insoluble forms in aging flies [24]. Because the disruption of the circadian clock does not affect the pathogenicity of these peptides, we assume that it has no effect on Aβ aggregation or clearance. In sum, our data show that the molecular and behavioral arrhythmia characteristic for *per*-null flies is not detrimental in this AD fly model.

However, our study shows that associations between AD and altered behavioral rhythms, observed in humans and AD model mice, also extend to fly AD models. Pan-neuronal expression of Aβ_42_ caused age-dependent impairment of circadian rest/activity rhythms, such that a reduced fraction of 50-days old *elav>Aβ_42_* flies remained rhythmic in constant darkness compared to controls. A more dramatic disruption of circadian rhythms was observed in *elav>Aβ_42_arc*. In LD, 5-day old flies of this genotype showed bimodal activity with an attenuated morning activity peak, while no activity peaks were detected in 15-day old flies, rather they were active around the clock, including nighttime when control flies had prolonged rest. While our work was nearing completion, another report that investigated links between AD and circadian rhythms was published [25]. The authors also found a loss of locomotor activity rhythms in *elav>Aβ_42_arc* flies even at young age, similar to our findings. Together, these results demonstrate that AD model flies have rest/activity rhythm degradation reminiscent of the behavioral degradation observed in humans with AD.

Loss of rest/activity rhythms in *elav>Aβ_42_arc* flies prompted us to investigate the functional status of central pacemaker neurons, which are necessary and sufficient for the activity rhythms, at least in young flies. Immunocytochemistry of PDF-positive pacemaker neurons sLN_v_ and lLN_v_ showed the correct number and arborization pattern in *elav>Aβ_42_arc* flies. Moreover these neurons showed nuclear peak and trough of the core clock protein PER indistinguishable from wild type flies. Similar observations were published recently [25], and the authors additionally showed that even expression of the more pathogenic tandem Aβ_42_ construct [26] left molecular oscillations in pacemaker neurons intact [25]. Together, these data show dissociation between functioning molecular pacemaker and disrupted circadian coordination of rest/activity rhythms. This suggests that behavioral rhythm degradation observed in humans and mouse AD models may occur despite the presence of a functional central clock. Importantly, strong body temperature rhythms have been reported in AD patients [27] again suggesting that the central clock may be intact in AD. This is reminiscent of the situation in very old flies and mammals, which show degradation of rest/activity rhythms while their central pacemaker neurons continue to show molecular oscillations [28], [29].

While AD-related degradation of behavioral rhythms is not caused by malfunction of the central clock, other contributing factors remain to be investigated. Aβ related arrhythmicity might be due to non-cell-autonomous toxicity as focused expression of toxic peptides in clock containing cells does not affect behavioral rhythmicity, but expression outside of the pacemaker neurons may affect their synaptic connections [25]. Additionally, downstream neuronal or humoral output pathways leading from the central pacemaker network to the motor centers could be adversely affected by Aβ aggregates. For example, recent studies reporting a direct measurement of neuronal activity in *elav>Aβ_42_arc* flies revealed increased latency and decreased response stability of the pathways leading from the giant fiber system in the brain into motor neurons of the thoracic ganglia [30]. It is possible that neuronal deficits of this kind could disable output pathways from the central clock leading to fragmented rather than consolidated sleep. This may lead to a vicious cycle as sleep deprivation was shown to increase amyloid peptides in mice [31] and Aβ aggregation disrupts the sleep/wake cycle [12]. As flies provide a powerful toolkit to study both AD [32] and circadian rhythms [1], studies at the intersection of chronobiology and AD should help to provide insights into the mechanisms underlying AD-related pathologies.

## Supporting Information

Table S1 **Statistical analysis of climbing data by two-way ANOVA with Bonferroni's post-hoc test.**
(XLS)
